# A Pragmatic Application of Ultrasonography for the Assessment of Disease Activity in Patients with Early Inflammatory Arthritis

**DOI:** 10.3390/jcm10020283

**Published:** 2021-01-14

**Authors:** Seoung Wan Nam, Taeyoung Kang

**Affiliations:** 1 Department of Rheumatology, Wonju Severance Christian Hospital, Yonsei University Wonju College of Medicine, Wonju 26426, Korea; namsw@yonsei.ac.kr; 2 Kang Taeyoung Internal Medicine Clinic, Seoul 04701, Korea

**Keywords:** ultrasonography, disease activity, early inflammatory arthritis

## Abstract

The aim of the study was to examine the usefulness of targeted musculoskeletal ultrasonography (MSUS) in assessing the disease activity of patients with early inflammatory arthritis (EIA). Twenty-eight patients with EIA were enrolled. The MSUS examination of joints with arthritic signs (tenderness or swelling), measurement of 28-joint Disease Activity Score (DAS28), and its components were performed at four-week interval visits until power doppler (PD) US remission was achieved. Various MSUS parameters of grey scale (GS) and PD synovitis were measured. Pearson or Spearman correlation coefficients were determined for the purpose of the study. Data were gathered from a total of 85 visits. The Sum of GS grade correlated better with physical examination findings, while the Sum of PD grade correlated better with serum inflammatory markers and patient global health. However, Global OMERACT-EULAR Synovitis Score (GLOESS), which reflected both PD and GS grades, correlated evenly well with each clinical parameter. In addition, GLOESS correlated best with DAS28 in the overall study population (*p* < 0.01). Conclusively, our targeted MSUS parameters of arthritic joints, especially sums of semi-quantitative grades of synovitis, could be useful in monitoring patients with EIA.

## 1. Introduction

Musculoskeletal ultrasound (MSUS) is increasingly and widely used in various clinical settings for evaluating and monitoring patients with inflammatory arthritis [1]. Previous studies have shown that MSUS is a useful diagnostic tool for the early detection of synovitis as well as for predicting the development of rheumatoid arthritis (RA) in patients with early arthritis [2,3,4]. MSUS can also be utilized in assessing the prognosis of early arthritis by predicting the progression of future radiographic damage [5,6]. In addition, MSUS synovitis activity parameters were shown to be valid measurements for monitoring treatment responses in RA patients [7,8]. Moreover, US can differentiate between intra-joint synovitis and other peri-joint causes of pain/swelling including tenosynovitis, bursitis, and other soft tissue lesions [9,10]. Therefore, the addition of properly targeted US assessments would help improve the diagnosis and treatment of patients with suspected or established RA in daily clinical practice [10]. However, controversies still remain on how many and which joints should be assessed by which US index to evaluate inflammatory arthritis [11,12,13,14].

Inflammatory arthritis is the characteristic feature of rheumatic diseases. Moreover, RA is one of the most detrimental diseases among them that is characterized by chronic synovitis and subsequent joint destruction [15]. In some patients with early inflammatory arthritis (EIA), the term “undifferentiated arthritis” (UA) is used to describe patients whose disease cannot be clearly classified and differentiated from other diseases [16]. In studies of early arthritis cohorts, approximately a quarter to a third of patients were classified as UA at the initial evaluation, and 13–54% of them eventually progressed to RA (with RA defined according to the 1987 American College of Rheumatology (ACR) criteria) after 1–2 years of follow-up [15]. Current treatment strategies for RA emphasizes the recognition and treatment of EIA in order to reduce and prevent the risk of further joint damage and disability. According to the European League Against Rheumatism (EULAR) recommendation for the management of early arthritis, US could be utilized as a supplemental imaging tool for detecting arthritis, predicting prognosis, or making management decisions [16]. To date, there have been many clinical studies using MSUS in assessing disease activity in patients with established RA [17]. MSUS studies scarcely evaluated disease activity in patients with early RA or UA that physicians could easily encounter in daily clinical practice [18,19,20]. Therefore, we focused on measuring the activity of synovitis in patients with EIA applying a pragmatic MSUS approach by limiting target joints only to those with arthritic signs. We then examined the relationships between the pragmatic US parameters and the various disease activity parameters of inflammatory arthritis to identify if these US parameters properly reflect the disease activity in patients with EIA.

## 2. Materials and Methods

### 2.1. Patients

This study prospectively recruited 32 consecutive patients with arthritic symptoms of less than 24 weeks duration from the outpatient clinic at the Department of Rheumatology of the Wonju Severance Christian Hospital, Wonju, South Korea, from 2014 to 2015. Arthritis patients were limited to those with definite clinical synovitis (at least one swollen joint) among the peripheral joints. US evaluations for this study were limited to the target joints with clinically evident arthritic signs (tenderness or swelling). All the patients were evaluated by MSUS at baseline, and those without power doppler (PD) or grey scale (GS) synovitis at the target joints were excluded from the study. The entire study population was divided into two groups: (1) patients diagnosed with RA at the patient enrollment or during the follow-up period according to the RA classification criteria (either the 1987 American College of Rheumatology (ACR) classification criteria or the 2010 ACR/EULAR classification criteria) [21,22]; (2) patients with UA.

The study protocol was approved by the Institutional Review Board of the Wonju Severance Christian Hospital, and informed consent was obtained from all the participants (IRB no. CR214004).

### 2.2. Variables and Outcome Measurements

MSUS examinations of target joints and measurements of the Disease Activity Score 28 (DAS28) and its components (tender joint count (TJC), swollen joint count (SJC), erythrocyte sedimentation rate (ESR), C-reactive protein (CRP), and visual analogue scale for patient global health both assessed by the patient and physician (VAS-GH-Patient, VAS-GH-Physician, respectively)) were performed at 4-week interval visits until the intraarticular PD signal completely disappeared in the target joints. This status without any PDUS signal in all the target joints was arbitrarily defined as PDUS remission status. DAS28 was measured by both the original version based on ESR (DAS28-ESR) and a subsequent version based on CRP (DAS28-CRP) [23,24]. In addition, we repeatedly assessed if the patients met the RA classification criteria (either the 1987 ACR classification criteria or the 2010 ACR/EULAR classification criteria) at every visit [21,22].

### 2.3. US Assessment

US examination was performed and repeated by the same EULAR musculoskeletal ultrasound level 2 expert. Scanning was performed using a General Electric (GE) Logiq e (General Electric Healthcare, Wauwatosa, WI, USA). The same preset was used in all the US examinations. For the US evaluation of synovitis at each joint level, semi-quantitative gradings were conducted for both GS synovitis (GS grade) and PD synovitis (PD grade). In addition, the quantitative measurements of PD synovitis (PD area) and the ratio between the PD area and the GS synovitis area were calculated (PD/GS ratio). In detail, the free hand Region of Interest (ROI) was drawn for the quantification of the area of GS synovial hypertrophy, and the area of positive power Doppler pixels within the ROI together with the ratio of the PD area and ROI were calculated with the GE Q Analysis software. All the semi-quantitative gradings of PD and GS synovitis were conducted in accordance with the scoring system proposed by the Outcomes Measures in Rheumatology—EULAR (OMERACT-EULAR) US Task Force that ranged from 0 (absence of inflammation) to 3 (worst score) [8,25]. In addition, the OMERACT-EULAR composite US scores were assessed, which were the highest score of GS and PD grades for the individual joint [8,26]. Global US synovitis scores were used to reflect the activity of US synovitis at the patient level, the sum of measurements of each US parameter (PD grade, PD area, GS grade, PD/GS ratio, and EULAR-OMERACT composite US score) from every target joint: Sum of PD grade, Sum of PD area, Sum of GS grade, Sum of PD/GS ratio, and Global OMERACT-EULAR Synovitis Score (GLOESS) [8].

### 2.4. Statistical Analysis

For statistical analysis, data were expressed according to the properties of the variables. Continuous variables were presented as the mean and standard deviation. Categorical variables were presented as the frequency and percentage. For comparisons between the two groups, we conducted independent t-tests, chi-square tests, or Fisher’s exact tests as deemed appropriate. The relationships between each MSUS parameter and the relationships between MSUS parameters and clinical disease activity parameters were evaluated by Pearson’s correlation coefficients or Spearman’s correlation coefficients as appropriate. A *p*-value less than 0.05 was considered statistically significant, and all statistical analyses were performed using the Statistical Package for Social Sciences (SPSS) version 21.0 for Windows (SPSS Inc., Chicago, IL, USA).

## 3. Results

### 3.1. Patient Characteristics

**Figure 1:**
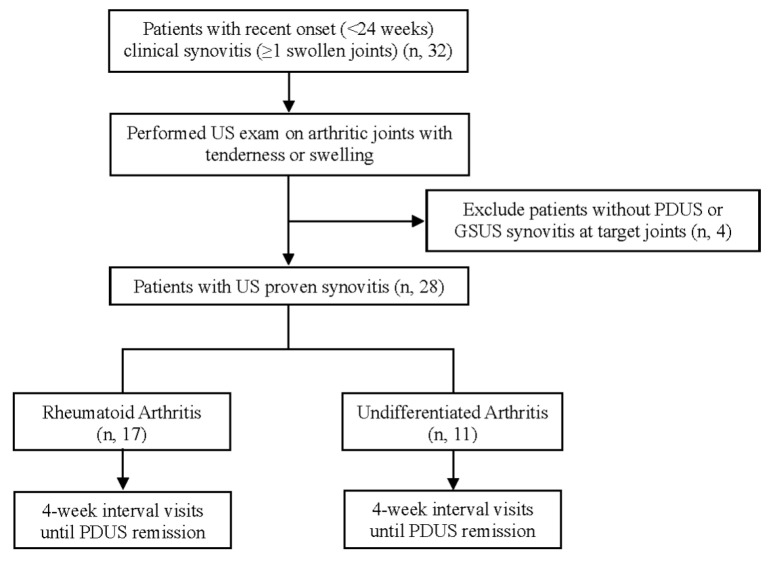
Patient selection flow. US, Ultrasonography; PDUS, Power doppler ultrasonography; GSUS, Grey scale ultrasonography.

Among the 32 patients who presented with clinical synovitis at patient enrollment, a final total of 28 patients were included in this study. Four patients without US proven synovitis were excluded from the study. A total of 60 and 25 visit data were collected until the PDUS remission in RA and UA patient groups, respectively. A total of 85 visit data points of clinical information and US evaluation results from the overall study population were gathered and analyzed in patients with EIA (Figure 1).

The demographic and clinical characteristics of the study population are shown in Table 1. The RA group of patients had more 4-week interval visits until PDUS remission, defined by the loss of positive PDUS signal, compared to the UA group (3.53 ± 1.97 in the RA group vs. 2.27 ± 0.79 in the UA group, *p* = 0.03). The proportions of patients with either positive immunoglobulin M-rheumatoid factor or anti-citrullinated protein antibody (anti-CCP) in each group did not exhibit significant differences. Six patients (35.3%) exhibited neither rheumatoid factor (RF) positivity nor anti-CCP antibody positivity in the RA group (seronegative RA patients). In addition, there were five patients (45.5%) with either RF and/or anti-CCP antibody positivity in the UA group (45.5% with RF positivity and 27.3% with anti-CCP positivity). At the initial visit, there were 16 patients who met the RA classification criteria. One patient, initially classified with UA at baseline, was reclassified with RA on his fourth visit (3 months after the baseline assessment). Among all the clinical and US disease activity parameters that were evaluated, SJC was only significantly higher in the RA group according to the initial visit data (3.41 ± 1.84 in the RA group vs. 1.73 ± 0.65 in the UA group, *p* < 0.01).

### 3.2. Distribution of Target Joints Examined and Proportions of US Proven Synovitis

The distribution of target joints with arthritic signs and proportions of US proven synovitis are presented in Table 2. During the total of 85 visits to the clinic, a total of 176 peripheral joints were found to have clinically evident synovitis and evaluated by MSUS. Among those 176 target joints, the wrist joint was the most frequently evaluated target joint with clinical synovitis (45.5%). In addition, the prevalence of US proven synovitis of each target joint was also the highest at the wrist joint (92.5%). In RA patients, all clinical synovitis of the metacarpophalangeal (MCP) joints and toe interphalangeal (IP) joints were revealed to have US proven synovitis. The proportions of US proven synovitis among clinically proven synovitis were lower in the knee, shoulder, and hand proximal interphalangeal (PIP) joints compared to other joints (40.0%, 44.4%, and 44.4%, respectively).

### 3.3. Correlations Between MSUS Parameters

The correlations between the MSUS parameters are shown in Table 3. The Sum of PD area, which was the sum of the quantitative measurements of synovitis, did not correlate well with the sums of the semi-quantitative gradings of synovitis (Pearson’s correlation coefficients (*r*) ranged 0.33–0.42, *p* < 0.01). However, the sum of ratios between the PD area and the area of GS synovitis (Sum of PD/GS ratio) correlated well with the sums of the semi-quantitative gradings of synovitis (*r* ranged 0.69–0.72, *p* < 0.01). GLOESS correlated better with the Sum of GS grade than the Sum of PD grade (*r* = 0.98 and 0.79, respectively, *p* < 0.01 each).

### 3.4. Correlations Between MSUS Parameters and Clinical Disease Activity Markers

Using the data from the 85 total visits, we evaluated the strength of the correlations between the MSUS parameters of synovitis and clinical disease activity markers of inflammatory arthritis as shown in Table 4. In the overall patients with EIA, GLOESS exhibited the highest intensity of correlations with both DAS28-ESR and DAS28-CRP (*r* = 0.64 and 0.69, respectively, *p* < 0.01). The parameters based on the quantitative measurement of synovitis such as the Sum of PD area and the Sum of PD/GS ratio showed weaker intensity of correlations with DAS-28 compared to the semi-quantitative gradings of synovitis in patients with RA. Moreover, Sum of PD area showed weaker intensity of correlations with DAS28 compared to other MSUS parameters in patients with UA. Notably, the Sum of PD grade better corelated with serum inflammatory markers (ESR and CRP) and both subjective and objective assessment of patient global health (VAS-GH-Patient and VAS-GH-Physician) compared to the Sum of GS grade. In contrast, Sum of GS grade correlated better with the physical exam findings of SJC and TJC. Both the Sum of GS grade and GLOESS showed high intensity of correlations with SJC (*r* or spearman’s rho (*r_s_*) ranged 0.73–0.81, *p* < 0.01) and moderate intensity of correlations with TJC (*r* or *r_s_* ranged 0.49–0.59, *p* < 0.01) in both patients with RA and UA. GLOESS correlated evenly well with various clinical disease activity parameters compared to Sum of PD grade or Sum of GS grade.

### 3.5. Positive Disease Activity Findings at the PDUS Remission Status

As shown in Table 5, not all patients achieved complete disease remission status at the PDUS remission status. In accordance with the widely accepted cut-off value of DAS28 < 2.6 in defining the clinical remission of RA, 32.1% or 7.1% of patients did not reach disease remission by DAS28-ESR or DAS28-CRP, respectively, at the PDUS remission status. However, all of them belonged to the low disease activity status of RA defined by DAS28 of ≤ 3.2 [27]. The median value for DAS28-ESR and DAS28-CRP were 2.49 (interquartile range (IQR) 1.82–3.10) and 1.64 (IQR of 1.36–2.06), respectively, at the PDUS remission status. Among all the evaluated clinical parameters, both ESR and DAS28-ESR most frequently exhibited values representing positive disease activity status at the PDUS remission status (32.1%).

## 4. Discussion

In the current study, we demonstrated the relationships between various MSUS parameters focusing on target joints with arthritic signs (tenderness or swelling) and clinical disease activity markers in patients with EIA. The unique features of the current study include that we introduced pragmatic MSUS evaluation methods by focusing on arthritic joints instead of assessing regular set of joints. Those MSUS parameters were not confined to the semi-quantitative gradings of synovitis (Sum of PD grade, Sum of GS grade, and GLOESES), but also included the quantitative measurements of synovitis (Sum of PD area, Sum of PD/GS ratio). Various MSUS parameters were evaluated in a single cohort of patients of this study. In addition, in contrast to most of the previous relevant studies, we examined patients with EIA, instead of limiting target population to patients with established RA. Our study results revealed that our targeted MSUS disease activity parameters, especially sums of semi-quantitative grades of synovitis, correlated well with various disease activity markers. In addition, our results showed how differently each MSUS parameter correlated with each clinical disease activity marker. Our key findings validate the targeted pragmatic application of MSUS in assessing the disease activity of EIA using less time and effort.

We included patients with recent onset (<24 weeks) clinically evident synovitis who also exhibited US proven synovitis at targeted arthritic joints. The definition for clinical synovitis, adopted from the 2010 ACR/EULAR criteria for RA, is having at least one swollen joint upon physical examination [22]. The presence of synovitis indicates the presence of inflammatory arthritis. In clinical practice, the assessment of early synovitis can be challenging even for experienced physicians. Therefore, MSUS has been recommended as an imaging tool that can be used to confirm synovitis supplementing clinical exam findings [15,16]. During the study period, all patients received routine medical care at the discretion of their rheumatologist. Even though there was no significant difference in the use of medications, patients with RA required more regular visits until achieving PDUS remission as shown in Table 1. Since the positive PDUS signal in joints signifies active synovitis, this result indicates that the activity of synovitis in early RA patients was less responsive to the conventional drug therapy compared to patients with UA in this study [28,29,30,31,32,33]. The prevalence of clinical synovitis was higher in RA patients compared to UA patients exhibiting higher SJC (*p* < 0.01). In addition, the prevalence of US proven synovitis in target arthritis joints was also higher in patients with RA as shown in Table 2 (79.4% in RA patients vs. 57.8% in UA patients, *p* < 0.01). The subjectivity of physical examinations, variable origins of tenderness including extraarticular structures, or the presence of joint effusion without synovial hypertrophy may explain this discrepancy between physical examinations and MSUS findings [33]. Although we did not perform an extended survey to confirm the final diagnoses in patients with UA, the prevalence of RF and/or anti-CCP antibody positivity was much higher in this group compared to the prevalence in the general population ascertained from previous studies (4.3% for RF and 0.8% for anti-CCP antibody in the general population) [34,35]. Therefore, many of the patients in UA group could have the potential to develop RA [34,36]. The phase of EIA, regardless of early established RA or UA, is the critical period that clinicians could halt disease progression through the early initiation of effective treatment [15,37]. Therefore, investigating the role of MSUS in monitoring EIA must be clinically meaningful.

Most previous studies concentrated on validating the use of MSUS through the routine examination of a fixed set of joints in patients with established RA [1,2,3,6,7,13,33,38,39]. Depending on the complexity of the US evaluation methods, time constraints could limit their application in daily clinical practice. In this respect, several recent studies attempted to minimize the number of joints assessed in monitoring the activity of RA [33,40]. However, it is still possible that the activity of synovitis in the remaining non-targeted arthritic joints could be ignored in their assessments of disease activity. Yoshimi et al. investigated whether on-demand MSUS on the most symptomatic joint alongside the routine US examination of eight joints is useful for detecting RA synovitis [41]. According to their study results, additional US exams on the most symptomatic joint detected synovitis in 38% of patients who reported to have no synovitis from the examination of the regular set of eight joints. Therefore, it would be more reasonable to apply our pragmatic targeted MSUS evaluation methods in the evaluation of the activity of inflammatory arthritis. Most of the relevant previous studies focused on quantifying the disease activity of RA using the sums of the semi-quantitative gradings of synovitis with PDUS and/or GSUS [33,40,42]. Only a few studies have evaluated the quantitative US measurements of synovitis in the assessments of disease activity. Moreover, this is the first study to demonstrate correlations between MSUS parameters using quantitative measurements of synovitis and clinical disease activity markers [40]. According to our study results, the sum of quantitative measurements did not correlate better with disease activity markers compared to the sum of the semi-quantitative indices in the overall patients (Table 4). MSUS parameters based on the semi-quantitative grading of synovitis were more accurate and less time consuming in assessing the disease activity of EIA compared to those based on quantitative measurements in this study.

GS ultrasonography (GSUS) and PDUS are two representative US imaging methods that assess the different components of synovitis [8]—synovial hypertrophy/effusion by GSUS and synovial vascularization by PDUS [33]. Between the two representative semi-quantitative MSUS synovitis markers, the Sum of GS grade correlated better with physical examination findings such as SJC or TJC. The Sum of PD grade correlated better with serum inflammatory markers (ESR and CRP) and assessments of patient global health (VAS-GH). All three previous studies that evaluated the correlations between MSUS parameters and clinical disease activity markers have shown that the sums of the PDUS or GSUS semi-quantitative grades correlated well with DAS28 in patients with established RA [33,42,43] and patients with EIA [19]. However, the sums of the GSUS grades did not exhibit significant correlations with DAS28, unlike the sums of the PDUS grades, in a study of 50 RA patients reported by El-Gohary et al. [33]. GSUS examination is useful in differentiating between joint effusion and synovial hypertrophy, but not useful in differentiating between acute and chronic synovitis. Synovial hypertrophy was regarded as evidence of disease activity but could also be the result of chronic inflammation without current active inflammation [42]. In contrast, PDUS is a useful US method to confirm active synovitis in target joints. In this context, many studies including the current study have demonstrated that the sum of the PDUS grades correlate with serum inflammatory markers and SJC in patients with RA [33,42,43]. However, the Sum of PDUS grade did not correlate with TJC in patients with RA in this study. Instead, the Sum of GSUS grade correlated better with TJC and also with SJC in all patient groups indicating that GSUS parameters could compensate for PDUS parameters in assessing the disease activity of inflammatory arthritis. Teresleve et al. demonstrated that the improvement in synovial hypertrophy during treatment was even greater in joints with GS synovial hypertrophy without PDUS activity compared to joints with GS synovial hypertrophy with PDUS activity in RA patients. Therefore, they concluded that synovial hypertrophy without PDUS activity should also be included in the assessment of disease activity by MSUS [44]. Moreover, as shown in Table 5, a significant proportion of patients with PDUS remission status still presented at least some degree of positive disease activity based on other disease activity parameters. These results indicate that the disease activity of inflammatory arthritis cannot be fully evaluated with PDUS alone. According to our study results, GLOESS, which reflects both the intensity of the PD signal and the extent of GS synovitis of the target joints [8], correlated evenly well with all the clinical parameters evaluated in this study. In addition, GLOESS correlated best with the composite disease activity score, DAS28, in the overall patients with EIA (*p* < 0.01).

Our study findings reveal that targeted MSUS parameters focusing on arthritic joints could be useful in assessing the disease activity of EIA. This approach could be especially helpful in busy clinical settings by saving more time. Both sums of semi-quantitative grades of PDUS and GSUS of synovitis seem to reflect the disease activity well in patients with EIA, but each with different characteristics. GLOESS, which reflects both characteristics of the GSUS and PDUS evaluation of synovitis, could be useful in the assessment of overall disease activity in patients with EIA. Our study has some limitations including a small sample size. Though the current study was an observation study representing simple correlations between two variables, our results could have been biased by the patient characteristic variables, to some extent, owing to its small sample size. In addition, it would have been better if we also compared results with MSUS evaluations of regular set joints. Further studies are necessary in the application of MSUS for monitoring patients with EIA.

## 5. Conclusions

Our targeted MSUS disease activity parameters of arthritic joints, especially the sums of semi-quantitative grades of synovitis, could be useful in monitoring patients with EIA.

## Figures and Tables

**Table 1:** Demographic and clinical characteristics.

Characteristics	Total Patients (n = 28)	RA Patients (n = 17)	UA Patients (n = 11)	*p*-Value ^a^
Sex—Female	20 (71.4%)	10 (58.8%)	10 (90.9%)	0.10
Age	56.43 ± 14.66	54.47 ± 14.86	59.46 ± 14.51	0.39
IgM-RF positivity	15 (53.6%)	10 (58.8%)	5 (45.5%)	0.39
Anti-CCP positivity	10 (35.7%)	7 (41.2%)	3 (27.3%)	0.69
At the initial visit				
met RA classification criteria	16 (57.1%)	16 (94.1%)	0 (0)	<0.01
Swollen joint count	2.75 ± 1.69	3.41 ± 1.84	1.73 ± 0.65	<0.01
Tender joint count	2.14 ± 2.16	2.59 ± 2.62	1.46 ± 0.82	0.11
VAS-GH-Patient	75.00 ± 15.52	76.47 ± 16.93	72.73 ± 13.48	0.54
VAS-GH-Physician	72.86 ± 13.84	75.29 ± 15.05	69.09 ± 11.36	0.25
ESR (≤20 mm/hr)	47.29 ± 27.33	48.65 ± 30.35	45.18 ± 23.10	0.75
CRP (<0.3 mg/dL)	3.03 ± 3.88	2.48 ± 3.28	3.88 ± 4.71	0.36
DAS28-ESR	4.77 ± 0.80	4.91 ± 0.83	4.56 ± 0.75	0.28
DAS28-CRP	4.18 ± 0.80	4.34 ± 0.83	3.95 ± 0.74	0.22
Sum of PD area	58.35 ± 66.01	61.69 ± 77.42	53.19 ± 46.11	0.75
Sum of PD/GS ratio	0.21 ± 0.25	0.23 ± 0.30	0.18 ± 0.14	0.65
Sum of PD grade	2.82 ± 2.18	2.76 ± 2.44	2.91 ± 1.81	0.87
Sum of GS grade	5.29 ± 4.36	6.35 ± 5.13	3.64 ± 2.06	0.11
GLOESS	5.64 ± 4.23	6.65 ± 5.04	4.09 ± 1.81	0.12
Number of visits until PDUS remission	3.04 ± 1.71	3.53 ± 1.97	2.27 ± 0.79	0.03
Medications used during the study period				
Steroids	28 (100%)	17 (100%)	11 (100%)	
Mean steroid dose (mg/day)	6.54 ± 3.75	6.49 ± 4.70	6.62 ± 1.14	0.93
Methotrexate	16 (57.1)	11 (64.7)	5 (45.5)	0.44
Sulfasalazine	12 (42.9)	8 (47.1)	4 (36.4)	0.71
NSAIDs	25 (89.3)	15 (88.2)	10 (90.9)	1.00

Continuous variables were presented as mean and standard deviation (mean ± SD). Categorical variables were presented as frequency and percentage. RA, Rheumatoid arthritis; UA, Undifferentiated arthritis; IgM-RF, Immunoglobulin M-rheumatoid factor; Anti-CCP, Anti-citrullinated protein antibody; VAS-GH, Visual analog scale-global health; ESR, Erythrocyte sedimentation rate; CRP, C-reactive protein; DAS28, Disease activity score 28; PD, Power doppler; GS, Grey scale; GLOESS, Global OMERACT-EULAR Synovitis Score; PDUS, power doppler (PD) ultrasonography; NSAIDs, Nonsteroidal anti-inflammatory drugs. ^a^ *p*-value for the comparison between RA patients and UA patients.

**Table 2:** Distribution of target joints with arthritic signs (A) and proportions (*p*) of ultrasonography proven synovitis (S) ^a^.

Target Joint	Total Patients (85 Visits)			RA Patients (60 Visits)			UA Patients (25 Visits)		
	A	S	*p* (%)	A	S	*p* (%)	A	S	*p* (%)
Shoulder	9	4	44.4	7	4	57.1	2	0	0
Elbow	7	3	42.9	3	2	66.7	4	1	25.0
Wrist	80	74	92.5	67	62	92.5	13	12	92.3
MCP	9	7	77.8	5	5	100	4	2	50.0
Hand PIP	9	4	44.4	7	4	57.1	2	0	0
Knee	20	8	40.0	15	8	53.3	5	0	0
Ankle	15	11	73.3	7	5	71.4	8	6	75.0
MTP	17	10	58.8	13	7	53.8	4	3	75.0
Toe IP ^b^	10	9	90.0	7	7	100	3	2	66.7
Total	176	130	73.9	131	104	79.4	45	26	57.8

^a^ Variables were presented as frequency unless otherwise indicated. RA, Rheumatoid arthritis; UA, Undifferentiated arthritis; MCP, Metacarpophalangeal joint; PIP, Proximal interphalangeal joint; MTP, Metatarsophalangeal joint; IP, Interphalangeal joint. ^b^ Toe IP included both 1st IP joints and 2nd to 5th PIP joints.

**Table 3:** Correlations between musculoskeletal ultrasonography parameters.

	Sum of PD Area	Sum of PD/GS Ratio	Sum of PD Grade	Sum of GS Grade	GLOESS
Sum of PD area	1	0.33 **	0.42 **	0.33 **	0.34 **
Sum of PD/GS ratio	0.33 **	1	0.72 **	0.70 **	0.69 **
Sum of PD grade	0.42 **	0.72 **	1	0.72 **	0.79 **
Sum of GS grade	0.33 **	0.70 **	0.72 **	1	0.98 **
GLOESS	0.34 **	0.69 **	0.79 **	0.98 **	1

Pearson’s correlation coefficients (*r*) are presented. ** *p* < 0.01. PD, Power doppler; GS, Grey scale; GLOESS, Global OMERACT-EULAR Synovitis Score.

**Table 4:** Correlations between musculoskeletal ultrasonography parameters and clinical disease activity markers ^a^.

Patient Groups	US Parameters		Disease Activity Parameters						
		SJC	TJC	VAS-GH		ESR	CRP	DAS28	
				Patient	Physician			ESR	CRP
Total patients (n, 85)	Sum of PD area	0.20	0.13	0.32 **	0.33 **	0.20	0.18	0.45 **	0.47 **
	Sum of PD/GS ratio	0.42 **	0.22	0.42 **	0.45 **	0.14	0.30 **	0.36 **	0.45 **
	Sum of PD grade	0.42 **	0.23 *	0.62 **	0.66 **	0.43 **	0.66 **	0.54 **	0.63 **
	Sum of GS grade	0.74 **	0.51 **	0.53 **	0.56 **	0.36 **	0.45 **	0.61 **	0.65 **
	GLOESS	0.74 **	0.50 **	0.57 **	0.61 **	0.42 **	0.52 **	0.64 **	0.69 **
RA patients (n, 60)	Sum of PD area	0.18	0.11	0.32 *	0.34 *	0.18	0.20	0.47 **	0.51 **
	Sum of PD/GS ratio	0.39 **	0.18	0.37 **	0.42 **	0.08	0.30 *	0.32 *	0.40 **
	Sum of PD grade	0.43 **	0.22	0.60 **	0.65 **	0.45 **	0.73 **	0.54 **	0.60 **
	Sum of GS grade	0.73 **	0.50 **	0.53 **	0.58 **	0.37 **	0.56 **	0.65 **	0.68 **
	GLOESS	0.73 **	0.49 **	0.57 **	0.62 **	0.44 **	0.65 **	0.67 **	0.71 **
UA patients ^b^ (n, 25)	Sum of PD area	0.57 **	0.47 *	0.44 *	0.46 *	0.24	0.32	0.40	0.52 *
	Sum of PD/GS ratio	0.68 **	0.53 *	0.61 **	0.66 **	0.35	0.54 *	0.55 **	0.68 **
	Sum of PD grade	0.62 **	0.51 *	0.68 **	0.73 **	0.48 *	0.72 **	0.65 **	0.81 **
	Sum of GS grade	0.80 **	0.56 **	0.59 **	0.61 **	0.23	0.42	0.49 *	0.64 **
	GLOESS	0.81 **	0.59 **	0.68 **	0.70 **	0.34	0.57 **	0.59 **	0.77 **

^a^ Pearson’s correlation coefficients (*r*) are presented unless otherwise indicated. ^b^ Spearman’s correlation coefficients (*r_s_*) are presented in this group. ** *p* < 0.01; * *p* < 0.05. US, Ultrasonography; SJC, Swollen joint count; TJC, Tender joint count; VAS-GH, Visual analog scale-global health; ESR, Erythrocyte sedimentation rate; CRP, C-reactive protein; DAS28, Disease activity score 28; PD, Power doppler; GS, Grey scale; GLOESS, Global OMERACT-EULAR Synovitis Score; RA, Rheumatoid arthritis; UA, Undifferentiated arthritis.

**Table 5:** Patients with positive disease activity findings at the PDUS remission status ^a^.

**Positive Disease Activity Findings**	**Number of Patients** **(n = 28)**
Swollen joint count ≥ 1	6 (21.4)
Tender joint count ≥ 1	4 (14.3)
VAS-GH-Patient > 10	4 (14.3)
VAS-GH-Physician > 10	4 (14.3)
ESR > 20 mm/hr	9 (32.1)
CRP ≥ 0.3 mg/dL	7 (25.0)
DAS28-ESR ≥ 2.6	9 (32.1)
DAS28-CRP ≥ 2.6	2 (7.1)
Sum of GS grade or GLOESS ≥ 1	10 (35.7)

^a^ Variables were presented as frequency and percentage. PDUS: Power doppler ultrasonography; VAS-GH: Visual analog scale-global health; ESR: Erythrocyte sedimentation rate; CRP: C-reactive protein; DAS28: Disease activity score 28; GS, Grey scale; GLOESS, Global OMERACT-EULAR Synovitis Score.

## Data Availability

The data presented in this study are available on request from the corresponding author. The data are not publicly available due to the ethical issues.
